# Novel Use of a Porcine Bladder Extracellular Matrix Scaffold to Treat Postoperative Seroma in a Total Knee Arthroplasty Patient

**DOI:** 10.1016/j.artd.2020.12.007

**Published:** 2021-01-30

**Authors:** Patrick Bettiol, Cameron Cox, Chris Gerzina, Jordan Simpson, Brendan MacKay

**Affiliations:** Department of Orthopaedic Surgery, Texas Tech University Health Sciences Center, Lubbock, TX, USA

**Keywords:** Seroma, Revision total knee arthroplasty, Extracellular matrix, Urinary bladder matrix

## Abstract

Seroma formation in a knee arthroplasty surgery is a rare complication. When seromas occur, they act as a nidus for bacterial growth and create an optimal environment for surgical site infections. In this case report, a 52-year-old woman presented with a seroma after multiple revision operations on the left knee. Owing to multiple failures of standard irrigation and drainage procedures to resolve the seroma, an orthoplastic colleague was consulted. Over five-and-a-half months, the patient underwent multiple procedures that failed to treat the seroma. However, in a final exploratory procedure, 3000 mg of urinary bladder matrix and negative pressure wound vacuum were placed. Seven months after the intervention, the patient had complete resolution.

## Introduction

A seroma occurs when an anatomical “dead space” fills with blood or serous transudate. These often occur when a wound is approximated and requires significant healing of a subcutaneous defect [1,2]. Although a seroma is comprised of sterile fluid, it can act as a nidus for postoperative infections as it can delay the wound healing process, leaving the site vulnerable to bacterial colonization [[2], [3], [4], [5], [6], [7], [8]]. Postoperative seroma is most common in patients undergoing breast cancer surgery and abdominal wall reconstruction because of the large amount of “dead space” beneath the skin and fascia caused by tissue removal [2,9,10]. Seromas are less prevalent in orthopedic surgery because most orthopedic procedures do not create large soft tissue defects in the subcutaneous and deep fascial layers. Within orthopedics, postoperative seroma occurs most frequently in patients who undergo soft tissue resection, skeletal tumor resections with large implant placement, lumbar spine surgery, cervical spine surgery, and hip arthroplasty [[11], [12], [13], [14], [15], [16], [17], [18], [19]].

Postoperative seroma is frequently treated by placement of a negative pressure incisional wound vacuum or drain placement [13,14,16,20]. Despite widespread use of negative pressure incisional wound vacuums as a treatment for seroma, a systematic review was inconclusive regarding efficacy of negative pressure wound therapy (NPWT) alone. The question remains whether NPWT reduces seroma recurrence compared with standard dressing [21]. Another systematic review and meta-analysis showed the utility of drains in prevention of seromas after axillary node dissections only, with no additional benefit in orthopedic procedures [22].

Given the lack of a gold standard algorithm for addressing a large postoperative “dead space” in this context, various biologic skin substitutes have come under consideration as potential adjuncts to assist in wound bed re-epithelialization. Acellular dermal matrices (ADMs) have been used in soft tissue defects to provide a low-immunogenic scaffold to promote epithelial tissue granulation and revascularization of native tissue [23]. ADM has been used extensively in abdominal wall and breast reconstruction [3,24]. While the majority of data show favorable outcomes in these procedures, some studies have indicated that ADM may not provide equivalent benefits in all surgical contexts [5,10,[24], [25], [26]]. Although a low-level immunogenic response is expected with ADM, some studies have observed a dense immunogenic reaction which slows the wound-healing process increasing the risk for postoperative complications [5,23,27].

Urinary bladder matrix (UBM) is a porcine ECM scaffold which not only maintains tensile and sheering properties of traditional ADMs but also permits epithelial cell penetration of the UBM by site-appropriate tissue, potentially mitigating the granulomatous-like reactions associated with ADM placement [28,29]. UBMs retain multiple intrinsic growth factors, including transforming growth factor-beta, vascular endothelial growth factor, platelet-derived growth factor, bone morphogenetic protein 4, and basic fibroblast growth factor, which directly influence wound healing [30]. Recent studies have also demonstrated antimicrobial properties of UBM to both *Staphylococcus aureus* and *Escherichia coli* as well as a predominance of macrophage phenotype 2 in the early postoperative period, which has been shown to reduce scar formation and enhance healthy functional tissue regeneration with increased chemotactic activity [31,32].

In patients with a postoperative seroma or large wound bed that requires a substantial amount of granulation tissue for “dead space” obliteration, UBMs provide a scaffolding to optimize organized tissue proliferation and potentially prevent further postoperative complications such as infection or dehiscence. In orthopedics, UBM use remains limited, and no cases have described the use of micronized UBM to treat recurrent seroma [[33], [34], [35]].

### Case history

A 52-year-old female presented to the clinic 6 weeks after primary bilateral total knee arthroplasty (TKA) with instability in her left knee. Medical comorbidities included: obesity (BMI: 38), systemic lupus erythematosus, fibromyalgia, and seronegative rheumatoid arthritis. Aspirate confirmed acute prosthetic joint infection (PJI) with staph epidermidis, and the patient underwent irrigation and drainage (I&D) with polyethylene exchange at 6 weeks after primary TKA. The left knee wound subsequently dehisced and repeat I&D with curettage was performed 10 weeks after primary TKA.

Seven and a half months after primary TKA, the patient presented with extensor mechanism rupture, patellar maltracking, TKA instability, arthrotomy dehiscence, and subcutaneous fluid collection. The patient had not been on any antibiotics for over 6 weeks and was scheduled for surgery. A thicker polyethylene liner was placed, and extensor mechanism reconstruction performed with 12 cm × 12 cm polypropylene mesh and FiberWire suture (Arthrex, Inc.; Naples, FL). Aspirate of the extra-articular effusion and intra-articular joint space were collected intraoperatively before reconstruction, and cultures, cell count with differential, and gram stain were ordered. Results showed no evidence of infection as defined by the Musculoskeletal Infection Society (MSIS) criteria for periprosthetic joint infection.

Three weeks after revision, the patient underwent a second repair for a 3-cm arthrotomy dehiscence, a persistent left knee effusion, and a re-rupture of the extensor mechanism. Upon exploration, an aspirate of the effusion was obtained, and the patella showed no signs of alta or baja and tracked appropriately on deep flexion and extension. The extensor mechanism dehiscence was repaired initially with a PDS absorbable suture and was subsequently reinforced by anchoring the previously placed mesh superiorly with a 12 cm × 6 cm propylene mesh with FiberWire suture to prevent new tearing. After completing the second extensor mechanism repair, the effusion specimen was cultured, gram stained, and found to be noninfectious.

**Figure 1:**
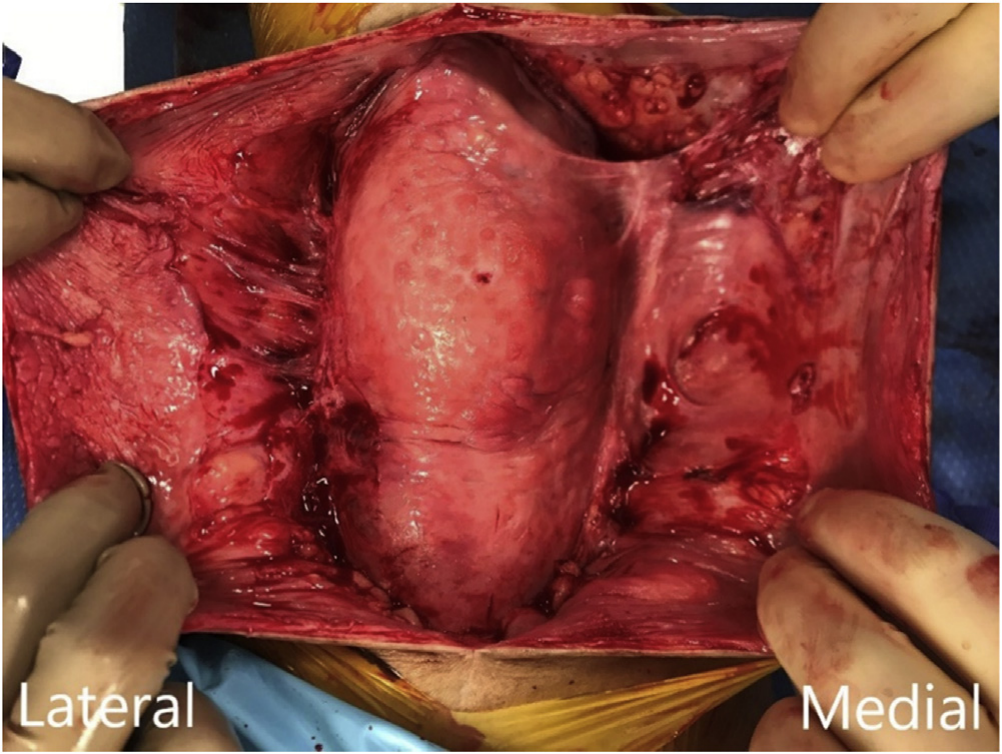
Intraoperative photograph with epithelialized tissue anterior to the capsule.

At 5 and a half months after revision, the patient again had a recurrent effusion over the left knee and was referred to an orthoplastic colleague for treatment. Serosanguinous transudate was drained from the extra-articular subcutaneous area, and seromadesis was performed. The fluid was located in a well-epithelialized pocket that was anterior to the pericapsular area and suspected to be a seroma (Fig. 1). Pathologic analysis with gram stain, cultures, and cell count with differential later confirmed that the fluid was not infected per the MSIS criteria. Furthermore, before the aspiration, the patient had not been on antibiotics since the perioperative antibiotics were given. The epithelialized tissue was dissected leaving a large space in the medial extraarticular space of the knee. To ensure adequate approximation of the subcutaneous tissue to the pericapsular area, excess skin and subcutaneous tissue was excised. Given the current algorithms for seromadesis, tetracycline and sterile talc powder were considered as potential sclerosants [36]. The decision was made to insert 16 grams of sterile talc powder into the medial extraarticular space with the intent to obliterate “dead space” and mitigate risk of seroma recurrence [36]. The incision was then closed, and a negative pressure wound vac was placed over the incision.

At 8 and a half months after revision and reconstruction, the seroma persisted with a fluid collection in the medial portion of the left knee extending longitudinally from the adductor tubercle to the distal portion of the medial tibial condyle. The patient had not been on antibiotics in over 2 weeks, and the seroma was aspirated. Subsequent culture, gram stain and cell count with differential of the transudate was negative for bacterial infection. C-reactive protein (CRP) and white blood cell (WBC) count were also within normal range consistent with a non-infectious process per the MSIS criteria. Surgery was planned for complete excision of the seroma with UBM placement. Intraoperatively, diagnosis of seroma was confirmed by a border of epithelialized tissue with no communicating sinus to the joint space. The seroma traveled ventral to the anterior capsule from the distal femur to the proximal tibia, and the wound bed measured 15 cm (L) × 5 cm (W). After excision, 1500 mg of UBM was placed in both the medial and lateral extraarticular space of the left knee, and a negative pressure wound vac was applied.

The wound was partially closed with an intermittent vertical mattress pattern to approximate the skin edges, and the remaining portion of the wound was closed with staples. The patient was discharged with a compression bandage to be worn for 6 weeks, a sealed incisional negative pressure wound vac to remain for 1 week, and a hinge knee brace locked in extension for 6 weeks.

At her 1-month postoperative follow-up visit, the patient reported pain and fluid accumulation, but no drainage from the incision site. Sutures and staples were removed, and aspirate was taken from the fluid accumulation. The patient had not been on antibiotics since the perioperative antibiotics were given. Aspirate cultures, cell count with differential, and gram stain were negative. The hematologic complete blood count differential, ESR, and CRP were within normal range consistent with a non-infectious process per the MSIS criteria.

**Figure 2:**
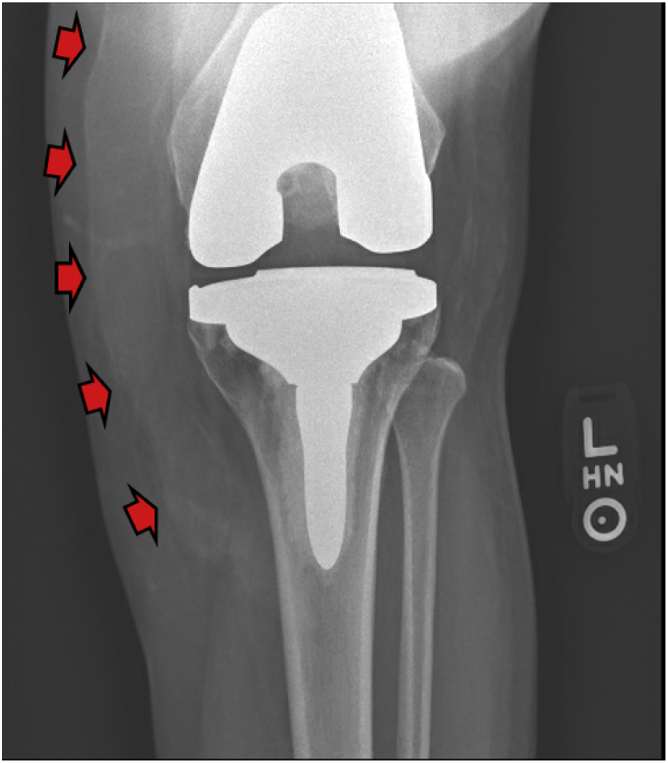
X-ray image showing swelling in the medial portion of the knee. Red arrows demonstrate effusion.

**Figure 3:**
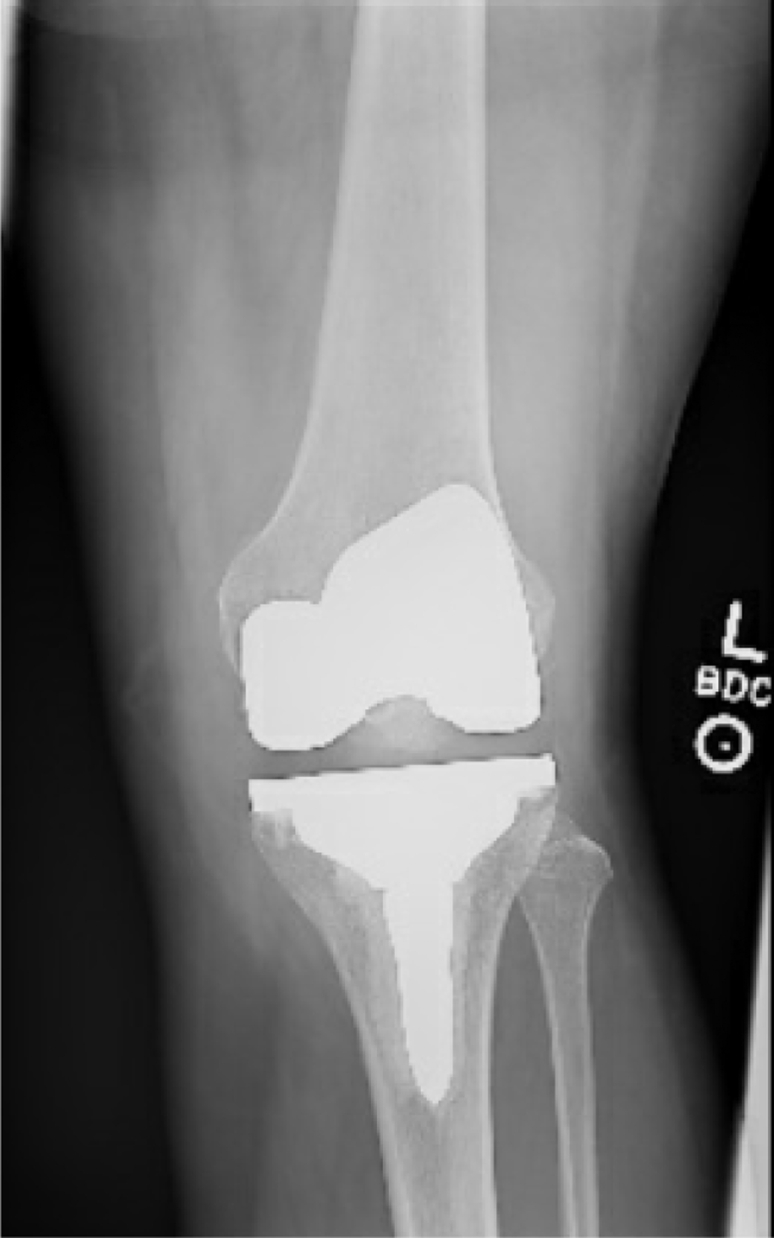
X-ray image showing resolved swelling in the medial portion of the knee 4 months after UBM application.

**Figure 4:**
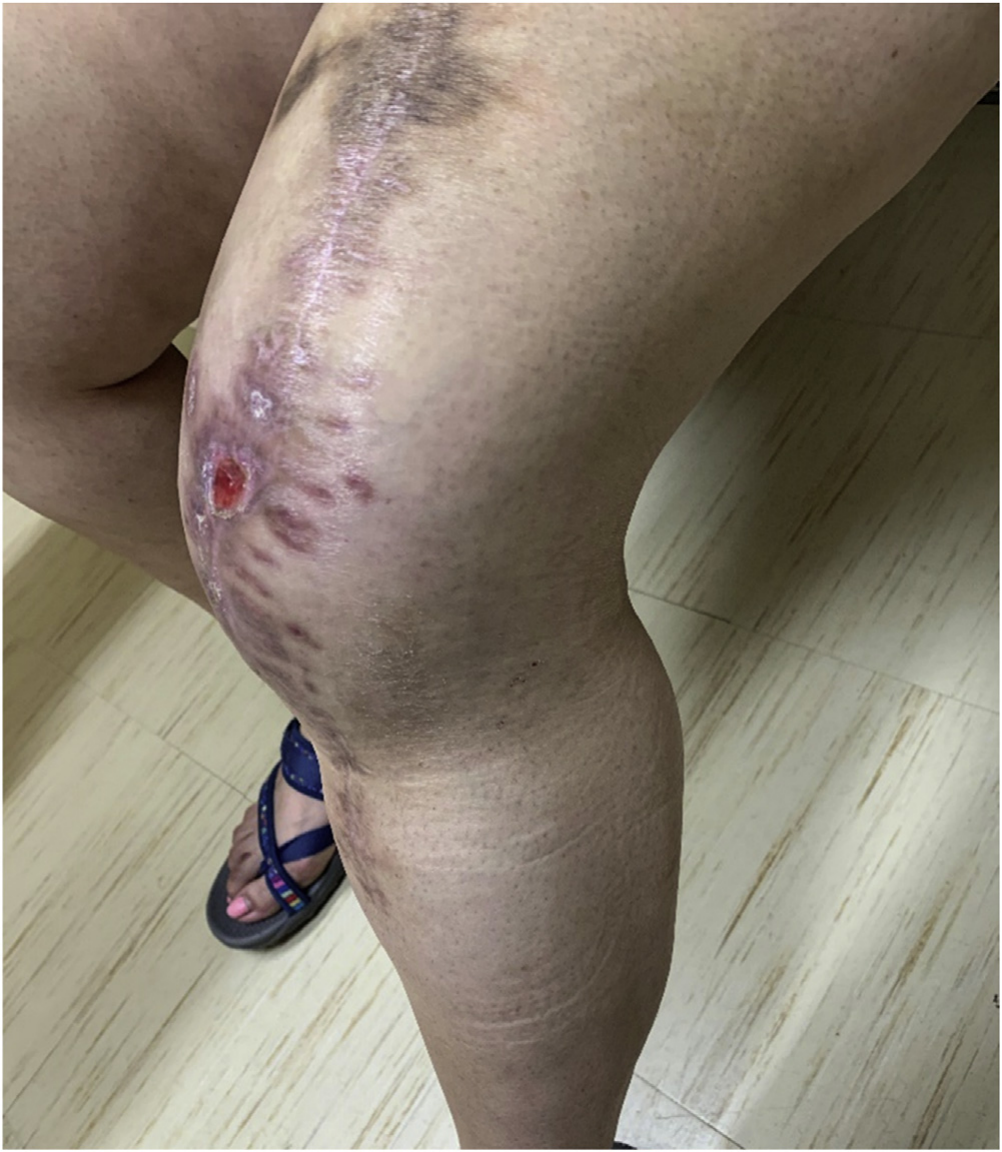
Clinical images showing near-complete healing of wound at 4 months after UBM application.

At 6 weeks after UBM placement, the patient presented to the clinic with transudative drainage and no pain. A scab was found on the medial aspect of the wound which the patient stated had been draining serous fluid. In clinic, the scab was deroofed, and serous drainage was observed. The patient then underwent formal I&D of the seroma. The wound bed had reduced from 15 × 5 cm to 9 × 4 cm with decreased fluid collection. Again, complete blood count differential was within normal range and culture, cell count with differential along with gram stain of the aspirate revealed a noninfectious specimen. The patient was discharged with a sealed incisional negative pressure wound vac, a compression bandage, and a hinge brace to remain locked in extension. The patient returned at a 4-month follow-up visit after the initial UBM placement and compared to radiographic images taken 2 weeks before micronized UBM placement (Fig. 2), there was limited swelling and the seroma appeared to have completely resolved both grossly and on radiographic images. (Figure 3, Figure 4).

Seven months after application of UBM, the patient underwent revision of all components and spacer placement for instability secondary to MCL insufficiency and posterior knee pain. Intraoperatively, the previously described dead space was found to be obliterated with no fluid collection. All follow-up appointments demonstrated no residual serous drainage and followed a similar postoperative strategy of locking hinge brace for 6 weeks, incisional wound vac for 1 week, and compression bandage for 6 weeks. After 2 subsequent I&D and polyethylene exchange procedures as well as external tissue expansion and wound vac placement, the wound was completely healed 1 year after UBM application with no concern for infection or residual sequelae.

## Discussion

Orthopedic procedures such as large bone resections, soft tissue resection, spine surgery, and hip arthroplasty may necessarily create an environment favorable to dead space formation between the deep fascial layer and subcutaneous tissue, which increases risk of seroma. In knee arthroplasty, however, there is little to no soft tissue between the capsule and the subcutaneous tissue which is what makes our case so rare. Large flaps were not raised during any of the procedures preceding seroma formation, and dissection was only performed to the minimum level required for excision of epithelialized tissue. While the exact mechanism is unclear, seroma has been shown to result from inflammatory exudates released with surgical insult to blood vessels, lymph vessels, and surrounding tissue [1,2,5,6,8,37]. In addition to the dead space created by TKA, our patient’s risk for recurrent seroma may have been amplified by irrigation and debridement procedures performed on the left knee before the first initial surgery. Furthermore, the literature has not demonstrated a relationship between pre-existing comorbidities or medications and increased the risk of seroma formation.

Micronized UBM was chosen over traditional ADMs in this case because of its intrinsic chemotactic factors known to promote epithelial cell penetration, nerve cell growth, and fibroblast migration, all of which aid in formation of granulation tissue to fill a large “dead space” [28,34]. Furthermore, given this patient’s history of PJI, a matrix with an associated antimicrobial mechanism was desirable. UBM has demonstrated antimicrobial activity against both *S. aureus* and *E. coli* [31]. After multiple failed treatments, UBM was the most viable option remaining that could potentially resolve the recurrent joint effusion while minimizing risk for PJI.

The successful obliteration of dead space using UBM in our patient may also be attributed to the differing intrinsic properties of UBM compared with ADM. Biopsies in breast reconstruction patients with ADM placement have shown lymphocyte, macrophages, granulocytes, and mast cell infiltrates that induced a foreign body and giant-cell reaction adjacent to the area of ADM [5,23,27]. This inflammatory mechanism may explain the increased incidence of seroma formation when using ADM reported in multiple studies [5,10,[24], [25], [26]].

Assessment of UBM alone in our case is confounded by the concurrent use of NPWT. As previously mentioned, there is ambiguity related to the efficacy of NPWT in the literature [21]. It is difficult to determine whether or not NPWT augmented the effect of UBM, or if the use of a wound vac or UBM alone could have achieved the same obliteration of dead space. However, one case series described 2 patients (one knee crushing injury and one breast mastectomy) in which initial NPWT-assisted closure failed [38]. In both patients, UBM was used in combination with NPWT to achieve granulation and eventual closure [38]. While these are not identical to the present case, results indicate that UBM could potentially improve outcomes compared to NWPT alone.

The current literature is limited in evaluating micronized UBM usage for orthopedic surgery; however, early reports indicate that it may have utility in this context, particularly in treating difficult open wounds and tendon repair [32,34,35,39]. Given the lack of clinical evidence surrounding UBM usage for treatment of seroma, our case may serve as the basis for future studies evaluating its efficacy in this setting.

## Summary

Although seroma formation in knee arthroplasty patients is rare, it presents a setback in the recovery process and can lead to infection. Although there is no simple solution for postoperative seroma management after knee arthroplasty, UBM may be considered as a treatment option in the setting of recurrent seroma in this patient population.

## Conflicts of interest

Although they are not directly related to this case report, the authors would like to disclose the following support for Dr. Brendan MacKay: Paid teaching for TriMed. Paid teaching and consulting, as well as research support, from AxoGen. Paid consulting for Baxter/Synovis and GLG. The authors declare there are no conflicts of interest.

## Supplementary data

Conflict of Interest Statement for Cox

Conflict of Interest Statement for Gerzina

Conflict of Interest Statement for Simpson

Conflict of Interest Statement for Bettiol

Conflict of Interest Statement for Mackay
